# Quality service in radiology

**DOI:** 10.2349/biij.3.3.e24

**Published:** 2007-07-01

**Authors:** J Hoe

**Affiliations:** Medi-Rad Associates Ltd, Mt. Elizabeth Medical Centre, Singapore

## INTRODUCTION

Radiology is part of the service industry and as a service provider one needs to understand quality and delivery of service. This includes knowledge of customer service, customer satisfaction and all its related issues as well as quality assurance and improvement issues.

Service quality and delivery in radiology are closely related but not the same. Nevertheless, both are required for customer satisfaction.

Academic and institutional radiology departments are now often considered as revenue centres in their hospitals. A cost centre is a unit or department in an organisation for which a manager is assigned responsibility for managing costs. Revenue generation is not the main concern of the department but cost control is of utmost importance. They are also often called support centres. With a revenue or profit centre, maximum revenues are generated and minimum expenses are incurred. The manager’s role is to maximise profit while minimising losses. Salary packages of academics also often include a clinical component. Senior radiologists need to have an understanding of the financial aspects as well as service aspects of a radiology department.

## THE DIFFERENCE BETWEEN SERVICE QUALITY AND SERVICE DELIVERY

Service quality and service delivery is not the same. Service quality is part of service delivery, and one of the components of service delivery.

Service quality can be defined as service that meets or exceeds the needs and expectations of a customer, making the customer happy. Good service quality is not the same as good quality control or quality assurance.

Good customer service can be defined as the ability of an organisation to constantly and consistently give their customers what they want and need, while excellent customer service means the organisation is able to constantly and consistently exceed the customer’s expectations.

Quality control is the process of checking on products (and sometimes services) to see if they meet requirements. This is usually carried out after the products have been manufactured. This concept does not usually apply to service industries like radiology departments. However, quality control does deal with some radiology issues such as acceptance testing of equipment and measurement of film processing parameters and calibration.

Quality assurance describes the introduction of documentation, and standardisation of quality systems and procedures to give customers confidence that the services would meet their expectations. Quality assurance means assurance of minimum quality standards, which are set by an external regulatory body and enforced through accreditation or certification combined with regular inspections.

It is achieved by designing processes and procedures, regularly checking and providing feedback and certification. Traditional quality assurance usually focuses on maintaining a quality level based on standards set by an external body e.g., ISO 9000-9002 standards, Joint Commission on Accreditation of Healthcare Organizations (JCAHO)-Joint Commission International (JCI) accreditation. Quality assurance helps to reinforce standards of care, and focuses on the individual provider as the source of poor outcomes.

Quality improvement is developed locally in response to the needs of the organisation or department. Quality improvement or continuous quality improvement (CQI) is a process that focuses on the processes of delivery of care and continuous improvement of performance indicators in radiology. These programs are part of total quality management (TQM), which is an approach that seeks to continuously improve every service that is provided and is an umbrella that covers all kinds of quality activities. It involves the concept of improving quality, rather than assuring it.

The essential elements of CQI are improving customer service; by breaking down the process into steps i.e., collecting and analysing the data; experimentation to test the implementation of ideas; and using teamwork to come up with solutions [1]. A good summary of quality was recently published [2,3].

The increased costs of implementing a quality program in a department may be offset by increased patient revenues or cost efficiencies elsewhere.

## WAYS TO IMPROVE SERVICE DELIVERY IN A RADIOLOGY DEPARTMENT

There are several basic steps in the process of improving service quality as well as service delivery in a radiology department.

**Table 1:** Quality Management in Radiology - definition of commonly used terms.

**Service Quality** meeting or exceeding the needs and expectations of the customer, delighting the customer and providing what he or she wants.
**Quality Control** process of checking on products to see if they meet requirements e.g., acceptance testing of equipment and daily film processing parameters.
**Quality Assurance** describes the introduction of, documentation and standardisation of quality systems and procedures to give radiologists and customers confidence that the services would meet their expectations. Achieved by designing processes and procedures, regularly checking, and providing feedback and certification e.g., ISO 9002, JACHO-JCI accreditation and Mammography Quality Standards Act (MQSA).
**Quality Improvement** focuses on the processes of delivery of care and is a concept of improving quality, rather than assuring it. Continuous quality improvement (CQI) focuses on improvement of performance indicators in radiology and seeks continuous improvement of every service. CQI programs are part of Total Quality Management (TQM), which is an umbrella that covers various quality activities.

### Understand how to identify the customer(s) of the department [4]

This is important, as only by identifying the customer, can the customers’ needs be determined and processes put in place to satisfy the customers. This may seem easy or intuitive as most would assume that the user of radiology services is the primary customer. However, it is important to know who are the primary customers and who are the secondary customers of the department. For an institutional radiology department, the customer could be a person, department or an organisation. This includes patients and their families, physicians, the hospital, hospital employees, other hospital departments, department employees as well as payers e.g., insurance companies, the federal or state government, Ministry of Health, etc. (Table 2).

**Table 2:** Customers of a Radiology Department.

**External Customers** Patients - inpatients, outpatients, their family members and relatives Physicians - referring physicians Other departments and their staff - wards and medical records Hospital administration and their staff - finance and corporate affairs Payers - insurance companies, Ministry of Health and Federal Government Regulatory agencies - radiation protection authority, etc.
**Internal customers** Radiology staff - receptionists, customer service officers, accounts staff, radiographers and managers Physicians - radiologists and nuclear medicine physicians

All these customers want different things e.g., patients want short waiting times, medical staff want fast and reliable reports, the hospital wants reliability and safety, the Ministry of Health or state/federal government usually want lower costs. Hospital administration also usually wants radiologists to provide fast, accurate and cost-effective services, and to keep patients and referring physicians happy. In an academic radiology department, the university administration is another customer, usually with different ideas about what constitutes effective service and performance – for example, education and research. In government and state-run and financed hospitals and departments, the main customer is the government which is mainly concerned about the cost of the services provided and compliance with regulations.

Often, the patient may not be the primary customer but is the one who has the potential to create most problems for a department if their demands are not met. A recent review has highlighted the importance of patients and the service to them [5]. Doctors tend to focus on patients as the primary customers but actually the referring physicians are probably more important so most departments should pay more attention to the needs of the referring doctors, as they should really be considered the primary customer of an institutional radiology department.

In private practice, the primary customer is the referring physician as he or she determines the need for the tests and refers the patient. It is the physician’s decisions that generate the demand for radiology examinations [6]. The patient is usually only the consumer of the service, hence is usually the secondary customer. However, as the secondary customer, the patient is also usually the person paying for the service unless payment is made by a third party e.g., private insurance, government subsidy or government reimbursement. Patients are sent to the department by their doctor and usually do not make the decision about where they go for their radiology examinations.

Customers can also be divided into internal or external customers e.g., doctors in the department (radiologists) and doctors from other departments in the hospital (referring physicians). The practice will likely already have many internal customers, that is people who are within the organisation. These people are those whom the radiologist serves and vice versa. The internal customers are also very important and all departments need to have training programs for them and empower them to provide efficient service to external customers. Employees who are happy in their work will focus more effectively on the external customers.

### Understand how customers assess radiology services

There are five main factors that determine customer satisfaction with radiology services [4]:

1. **Reliability** – ability to provide the service as promised to the customer and to do so accurately. In radiology, this means correct examination must be performed so that the correct views can be obtained. The report must be accurate and of high quality, regardless of who is reporting e.g., resident or consultant, and the report must answer the clinical problem of the patient.
2. **Responsiveness** – willingness and ability to help customers promptly. In radiology, this means being able to get appointments for patients quickly as well as sending the films and report soon after the examination to the referring doctor. Long waiting times for appointments and taking more than a couple of hours to generate an urgent report is generally not acceptable in most hospitals.
3. **Assurance** – the customer must feel comfortable with the competence of the service provider. Customers must get the feeling that they are receiving the best service and must have confidence in the service. In radiology, this means that the staff must not only be technically competent but must also have interpersonal skills, as they must be able to interact with both patients and referring physicians. Many radiologists do not bother to interact or talk to patients but the need for this interaction is growing, especially with interventional procedures.
4. **Empathy** – the radiologist needs to show some degree of caring and attention to customers. This again highlights the importance of interpersonal skills, which starts from front desk reception staff to the radiologist.
5. **Tangibles** – the physical appearance of the department and facilities, and the quality of the equipment. In radiology, because of high capital cost of equipment, it is not always possible to have the best equipment but it is always important that the available equipment is used correctly and the quality of work produced is of high quality. It is not advisable to take too many shortcuts to save money e.g., performing a couple of pulse sequences of a MRI scan and filming only a few images of the sequences on hard copy.

As discussed earlier, the main and primary customer of a private radiology department is the referring physician. Physician referral decisions are usually complex and influenced by a few main issues, such as the radiology practice’s professional quality; its helpfulness and support to the referring physician’s practice; its degree of personal convenience and familiarity to the referring physician, and its success at sending a satisfied patient back to the referring physician.

In a survey of referring physicians and the factors that make them refer patients to radiology groups in private practice, the most important factors in order of importance were found to be ability to schedule patients quickly; personal familiarity with radiologist; speed in receiving results as well as quality and accuracy of the radiologist [6] (Table 3). The two most important factors that made a referring physician change the radiology provider were delays in scheduling patients or in reporting.

**Table 3:** Most Important Factors in Selecting a Radiologist (modified after Lopiano *et al* [6])

**Factor**	**Percentage of respondents choosing**	
	**as most important factor**	**as one of top three factors**
Ability to schedule patients quickly	30%	68%
Personal familiarity with radiologist	29%	55%
Speed in receiving results	18%	58%
Convenient location	16%	43%
Range of techniques available	11%	37%
Hospital affiliation	8%	20%
Patient's preference	5%	18%
Others (quality & accuracy of radiologist*)	22%	27%

* should be regarded as one of top 3 factors as it was frequently specified.

Price of the radiology examination was not considered a major factor in the survey. In most developed countries, patients usually have their radiology examinations partly or fully paid for by a third party, such as insurance companies or the government. However, in many other countries, pricing of radiological exams is a major consideration as patients pay for their own investigations and referring physicians often only refer patients to the cheapest provider. It is important to make the physicians realise that the cheapest provider may not always be able to provide the best quality exams because of the lower cost of technology involved. In many countries, commissions are often also paid to the referring physician for sending a patient to the radiology practice. This practice is unethical as well as illegal, and it can also significantly affect the referral process. This serves to reinforce that the main customer of a private radiology provider is the referring physician.

Institutional radiologists should also realise that even referring physicians often have same feelings as private practice physicians about their department service, and would be willing to change service providers. In a study of preferences of users of institutional radiology services, which assessed the implications for retention and extension of impact on referral base, it was found that 33% of referring physicians would change their radiology service provider if their scheduling expectations were not met [7]. In another survey of referring physicians using CT and MR services of an academic radiology department, the main areas of concern were again long waiting time to get an appointment slot, long waiting time for the radiology exams to be performed and delays in getting the reports or lack of promptness for communication of important findings [8].

It is obvious again that long waiting time for radiology appointments is simply not acceptable even in an institution. The department should not assume that referring physicians in the hospital are captive customers and will always refer only to the department. For inpatients and interventional procedures, this is obviously not usually possible and they are forced to use the department, but it is also not uncommon for institutional physicians to refer their patients, especially fee paying or private patients, to another private radiology service provider.

### Understand how to identify customers’ needs and assess their satisfaction

Customers’ needs and expectations are always changing, therefore capturing or finding out their needs is a dynamic process. Appropriate questionnaires or surveys can be used to obtain accurate and reliable data on customers’ needs and expectations, and provide judgment of the quality of services.

Questionnaires need to be properly developed: they need to be short, easy to self-administer, questions must be quantitative and allow for multiple choices, and wording of questions is very important.

Radiologists should also measure the department logistics to get an idea of some of the many processes in the department that can cause problems that affect customer satisfaction. Possible starting points for improved service delivery are reduced patient waiting time in appointments for various radiology exams and reduced report turn around time.

Understanding the customer’s needs and expectations regarding quality of services is an essential part of improving service delivery. Various levels of customer’s expectations can be classified:

- **Basic assumed level of quality of service** – Physician expects to get a radiology report within 24 hours. The patient assumes that the correct radiology exam has been done.
- **Intermediate level of satisfaction** – Physician receives a written report within 24 hours but receives a phone call immediately after the exam when the exam reveals unexpected findings. The patient receives prompt attention at the reception desk and the examination is carried out quickly.
- **Highest level of expectation and customer satisfaction** – Patient receives prompt and courteous attention by the reception staff, is happy with the caring and courteous treatment during the examination and is pleased to receive an educational pamphlet regarding the exam while the referring physician was able to review his or her patient’s examination films and report within an hour after the radiology exam.

Good customer service has been defined as the ability of a department to constantly and consistently give the customer what they want and need while excellent customer service means the ability of a department to constantly and consistently exceed the customer’s expectation. If one were to be honest, most radiologists would probably agree that most institutional departments are barely able to provide good customer service.

A recommendation for institutions that want to improve their service delivery is to firstly clarify the mission of their department and then adopt a mission statement, so that the radiologist and the staff know the prime function of the department. Is it to provide research or to provide customer service and revenue? Then the institution should plan, adopt and implement this philosophy for the department. Next, a formal customer satisfaction program and team should be designed and implemented also in addition to pursuing certain regulatory status for the department e.g., ISO 9002, JCAHO-JCI accreditation, etc. Once these processes are in place, marketing of the radiology services to external and internal customers will be more effective [9].

It is important that the people working in the facility understand customer satisfaction and service delivery. Aside from proper training of the radiographers and support staff, it is important to identify the correct type of radiologists who are going to work in a private practice because having the adequate technical skills alone is not enough to be successful in the private sector. The radiologist needs to be able to interact with referring physicians, patients and colleagues much more than in an institutional or department setting where many responsibilities are delegated to residents and many radiologists do not want to talk to the patients or the referring physicians. Subspecialty-trained radiologists who are well-qualified and has recognized peer respect and patient-friendly skills in addition to being dynamic and outgoing, will always find employment easily in the private sector. The requirement for a successful practice is radiologists driven by service delivery.

Practice management indicators can also be used as tools to evaluate the department’s progress towards its goals. Performance movement indicators are objective tools that evaluate and assess key components of an organisation by setting performance goals and tracking performance over time [10]. In radiology, these indicators include categories such as productivity, finance, patient safety, access to examinations, radiology reporting, and customer satisfaction. Once specific indicators in each of these categories have been selected, data collection methods should be incorporated into the routine department processes. To improve the quality of service, these indicators should also be benchmarked. Most commonly used indicators in radiology reporting are report turn around time, transcription time and signature time. For customer satisfaction, the most commonly used indicators are patient complaints, patient satisfaction and patient waiting time. Referring physician satisfaction and employee satisfaction were used in less than half of the departments surveyed [11]. For productivity, the most commonly used indicator is examination volume while for financial status, general expenses is most commonly used. More specific performance indicators may be developed for the department so its performance can be better monitored.

## CONCLUSION

It is necessary for the radiologist to understand the difference between service quality and service delivery in radiology. Quality in service is only one component of service delivery in radiology. Knowledge of quality assurance in radiology and knowing how to implement and maintain a quality level based on standards set by an external body e.g., JCI, as well as knowing how to implement continuous quality improvement programs (part of TQM) in the department are also important [12]. All these processes and programs need to be implemented to ensure high quality in service and good service delivery to customers besides improving financial performance of the department. The increased costs of implementing a quality program in a department may be offset by increased patient revenues or cost efficiencies elsewhere.
